# Associations between physical activity levels and socioeconomic and occupational factors among a large workforce population: insights from an employee health cohort study

**DOI:** 10.1186/s12889-025-25957-2

**Published:** 2026-08-27

**Authors:** Parisa Nejati, Lida Nejati, Jamileh Abolghasemi, Seyed Abbas Motevalian

**Affiliations:** 1 Department of Sports and Exercise Medicine, Rasool Akram Hospital, School of Medicine, Iran University of Medical Sciences, Tehran, Iran; 2 Office of Executive Dean for Research, Miller School of Medicine, University of Miami, Florida, USA; 3 Department of Biostatistics, School of Public Health, Iran University of Medical Sciences, Tehran, Iran; 4 Preventive Medicine and Public Health Research Center, Psychosocial Health Research Institute, Iran University of Medical Sciences, Tehran, Iran; 5 Department of Epidemiology, School of Public Health, Iran University of Medical Sciences, Tehran, Iran

**Keywords:** Demographic status, Employees, IPAQ, Occupational status, Physical activity, Physical inactivity, Socio-economic status

## Abstract

**Background:**

Physical inactivity is a growing concern among working populations, with implications for health and productivity. Unlike most previous studies, this research draws on large-scale cohort data from Iranian employees to examine the associations with socioeconomic and occupational factors on physical activity, addressing an evidence gap in workforce health inequalities.

**Methods:**

This cross-sectional analysis used baseline data collected between 2017 and 2020 from the Employee Health Cohort Study of Iran (EHCSIR), a 15-year longitudinal project to identify risk factors of non-communicable diseases. EHCSIR participants are mainly employees affiliated with the Iran University of Medical Sciences and the Ministry of Health and Medical Education. Of the 4,886 EHCSIR participants, 3,997 with complete physical activity and sociodemographic data were included in the analysis. The data included employee age, gender, marital status, economic status (wealth index), education level, job, and workplace, calculated via principal component analysis (PCA). Physical activity level (i.e., mild, moderate, and vigorous) was measured using the short form of the International Physical Activity Questionnaire (IPAQ) in MET-minutes per week. It was also categorized according to the World Health Organization (WHO) guidelines, which recommend at least 150 min of moderate activity, 75 min of vigorous activity, or a total of 600 MET-minutes per week for adults. Accordingly, the employees were classified as physically inactive or adequately / highly active. The data were analyzed using multivariate logistic regression models together with fixed-effects and random-effects models.

**Results:**

Nearly 64% of employees in Iran did not meet the WHO’s recommendations for physical activity. The mean total physical activity level was 599.2 and 941.8 MET-minutes per week in female and male employees, respectively. The highest percentage of inactive employees was observed in 45-54-year-olds (66.3%), females (67%), married employees (64.7%), Q4 of the wealth index (70.1%), associates (66.2%), office workers (94.8%), and employees working in health centers (67.1%). Physical activity levels varied significantly across age, gender, job type, workplace, and socioeconomic status. Employees in sedentary roles and higher-SES groups were less likely to be physically active, while younger, male, and hospital-based staff showed higher activity levels.

**Conclusion:**

These findings reveal structural inequalities in workplace physical activity and underscore the need for targeted health promotion strategies. Several factors may contribute to insufficient physical activity among the participants, including low awareness about the benefits of physical activity, lack of workplace exercise facilities, the high cost of attending fitness classes and other related barriers. Interventions tailored to occupational context—such as PA-friendly environments and flexible scheduling—may help reduce inactivity and improve employee well-being.

## Introduction

Health inequality is a major concern in public health. As a personal or social determinant of health differences, physical inactivity has always been an issue of interest to researchers and health policymakers. The prominent role of physical inactivity in morbidity and mortality associated with chronic diseases [1, 2], on the one hand, and the high prevalence (27.5%) of physical inactivity [3], on the other hand, make it as important goal for WHO to increase the level of global physical activity [4].

Studies into public health have taken into consideration socio-demographic factors such as gender, age, race, and socioeconomic status (SES) as contributors to physical activity (PA) inequalities [5–11]. However, a review of the literature attests that the relationship between physical activity level (PAL) and socio-demographic status is not straightforward [10, 12–17].

Some recent studies show that PA inequality can be due to the PA domains. More specifically, low-SES groups are more active in terms of occupational and transportation PA whereas high-SES groups are more involved in leisure PA [6, 10, 16]. Indeed, determining the contributory factors in PAL can be helpful in health equality issues because some of these factors can be manipulated in order to improve PAL.

The existing literature indicates the negative impact of sedentary work on employees’ state of health and their job performance [18–22]. The PAL of employees has been suggested to have the potential to alter physical, cognitive, and affective resources with implications for a better performance [18, 20, 23–26].

While previous studies have examined general patterns of physical activity across demographic and occupational groups, there remains limited evidence on how these factors interact among employees in middle-income settings such as Iran. To address this gap, the present study explores inequalities in physical activity across different job types and socioeconomic strata within a large employee cohort. Based on prior evidence, we hypothesized that employees in lower socioeconomic strata and manual occupations would report higher physical activity due to job-related movement.

Accordingly, the following research questions and specific objectives were formulated.

### Research questions

What is the prevalence of adequate and high physical activity among employees?

How do sociodemographic characteristics (age, gender, marital status, education, and economic status) relate to physical activity levels?

How do occupational variables (job type and workplace) influence employees’ physical activity?

### Specific objectives

To quantify physical activity levels across demographic and occupational subgroups.

To determine the independent predictors of adequate and high physical activity using multivariate models.

To explore potential inequalities in physical activity related to socioeconomic and occupational status.

## Methods

### Study strategy

This cross-sectional analysis was conducted using baseline data between 2017 and 2020 from the Employee Health Cohort Study of Iran (EHCSIR), a cohort study of public-sector employees primarily working in schools, hospitals, and health centers affiliated with the Iran University of Medical Sciences (IUMS) and the Ministry of Health and Medical Education that began in July 2017. All of the permanent employees affiliated with the Iran University of Medical Sciences and the Ministry of Health were eligible to enter the cohort study. Among the 4,886 EHCSIR participants, 3,997 individuals who provided complete data on physical activity and sociodemographic questionnaires were included in the final analysis of this study. 

As this study was based on existing cohort data, no formal sample size calculation was performed.

All of the EHCSIR participants provided written informed consent. The study protocol was approved by the Research Ethics Committee of IUMS (Approval Code: IR.IUMS.REC.1399.219).

### Measures

#### Definitions

##### Jobs

- Faculty members: the faculty members (MDs or PhDs) staffing universities, hospitals, or other workplaces affiliated with the IUMS or Ministry of Health
- Healthcare professionals: employees such as physicians and nurses who provide health services at hospitals or other health centers related to the IUMS
- Office workers: employees working for the administrative departments of universities, hospitals, the Ministry of Health, or other parts of the IUMS
- Ancillary staff members: employees such as janitors, security guards, or nursing assistants who provide services in different parts of universities, hospitals, the Ministry of Health, or other sections of the IUMS

##### Workplaces

- Office: the administrative parts of universities or the Ministry of Health
- Health center: all health centers except hospitals that provide health services
- Hospital: all the hospitals affiliated with the IUMS

#### Sociodemographic data

Data were collected on the employees’ age (34; 35–44; 45–54; > 54 years), gender (female, male), marital status (married, unmarried, ex-married), job (faculty member, healthcare professional, office worker, ancillary staff member), workplace (office, health center, hospital), educational attainment (elementary, high school diploma, associate’s, bachelor’s, master’s, PhD) as well as wealth index. Wealth index was constructed using principal component analysis (PCA) based on ownership of 12 household assets: home ownership, car, motorcycle, refrigerator, washing machine, dishwasher, vacuum cleaner, television, cellphone, personal computer, internet access, and access to a landline phone. Each asset was coded as binary (0 = not owned, 1 = owned). The first principal component, which explained the largest proportion of variance (20%), was used to generate the index. The wealth index was measured by multiplying the coefficient of each factor in 0 (not having) or 1 (having). The respective factor scores were categorized into five quintiles: the lowest 20% of the wealth index was classified as having a very low economic status (Q1), the 21–40% as low (Q2), the 41–60% as medium (Q3), the 61–80% as high (Q4), and the highest 20% as very high (Q5). This method, adapted from Filmer and Pritchett’s approach [27], has demonstrated strong construct validity and is widely applied in national and sub-national studies in Iran [17]. The resulting wealth scores were categorized into five quintiles: Q1 (very low), Q2 (low), Q3 (medium), Q4 (high), and Q5 (very high).

#### Physical activity

Physical activity (PA) was measured using the short form of the International Physical Activity Questionnaire (IPAQ) [28]. The IPAQ assesses PA undertaken across a comprehensive set of domains, including leisure time, domestic and gardening activities, work-related activities, and transportation. The specific types of activity assessed in the present study were walking, moderate-intensity and vigorous activities, frequency (number of days per week), and time (per day). The total duration of PA in the previous week was computed by the aggregation of the time (in minutes) and frequency (days) of walking, moderate-intensity and vigorous activity.

To obtain a continuous scale, the amount of activity was scored in MET-minutes by multiplying the MET (Metabolic Equivalent of Task) score by total minutes. Walking, moderate-intensity PA, and vigorous PA were assigned MET scores of 3.3, 4.0, and 8.0, respectively. For categorical scoring, the WHO’s guidelines were used [29] which recommend adults should engage in at least 150 min of moderate-intensity activity, 75 min of vigorous-intensity activity, or a total of 600 MET-minutes per week to be considered adequately active. PA was considered adequate if an individual had at least 600 MET-minutes achieved through moderate or vigorous activities or at least 150 min of moderate-intensity activity, or at least 75 min of vigorous activity per week. Those who did not meet these criteria were classified as inadequately active. PAL was regarded as high if an individual reported at least 1200 MET-minutes achieved through moderate or vigorous activities, or at least 300 min of moderate activity, or at least 150 min of vigorous activity per week.

### Data processing

To fix and remove incorrectly formatted, duplicate, or incomplete data within the dataset of the EHCSIR, a three-member data cleaning team (1 MD and 2 PhDs) were employed. Once individuals with missing data on PAL and socioeconomic variables were excluded, data processing was performed based on the IPAQ guidelines, which were as follows:

- Time variables were converted from hours and minutes into total minutes to standardize measurement units and allow consistent calculation of MET-minutes per week, in accordance with the official IPAQ scoring protocol [28].
- The day variables of 8 or greater were accordingly excluded from analysis.
- The unreasonably high variables (at least 16 hours) were considered as outliers and were thus excluded from analysis.
- The time variables exceeding 4 hours or 240 minutes were truncated to be equal to 240 minutes

### Statistical analysis

Multivariate logistic regression models were applied to examine the association between physical activity status (adequate/inadequate and high/non-high) and sociodemographic and occupational variables. The outcome variable (physical activity status) was categorical, defined as adequate/inadequate and high /non-high activity according to WHO criteria.

Given the hierarchical structure of the data—with employees nested within workplaces—we applied mixed-effects models to account for potential clustering effects. Workplace was included as a random intercept in all models to capture unobserved heterogeneity across institutions. The intraclass correlation coefficient (ICC) was calculated to quantify the proportion of variance attributable to workplace-level clustering. In our models, ICC values ranged from 0.04 to 0.07, indicating modest but non-negligible clustering.

Model fit was assessed using Akaike Information Criterion (AIC) and Bayesian Information Criterion (BIC), and residual plots were examined to confirm assumptions of normality and homoscedasticity. All independent variables were entered simultaneously based on theoretical relevance; no stepwise selection procedures were applied. Adjusted odds ratios (AORs) and 95% confidence intervals (CIs) were reported for each predictor.

All independent variables—including age, gender, marital status, education level, wealth index, job type, and workplace—were entered into the models simultaneously. This approach was chosen to ensure that adjusted odds ratios (AORs) reflected the independent contribution of each variable while controlling for potential confounding effects. No variable selection procedures (e.g., stepwise regression) were used, as all covariates were retained based on theoretical relevance and prior evidence from the literature.

Total PA, expressed in MET-minutes per week, is presented as mean ± standard error (SE). To assess the associations between demographic and occupational variables and employees’ total PA levels, a mixed-effects general linear regression model was applied, accounting for potential clustering by workplace.

For statistical modeling, physical activity was coded as follows:

- In the first logistic regression model, “adequate PA” was coded as 1, and “inadequate PA” as 0.
- In the second model, “high PA” was coded as 1 if participants achieved ≥1200 MET-minutes/week, or ≥300 minutes/week of moderate activity, or ≥150 minutes/week of vigorous activity; otherwise, it was coded as 0.

These binary outcomes were used as dependent variables in multivariate logistic regression analyses to estimate adjusted odds ratios (AORs) and 95% confidence intervals.

Multicollinearity among predictors was assessed using variance inflation factors (VIFs). All VIF values were below 2.5, suggesting no significant collinearity between education, wealth index, or other covariates. A two-sided p-value of less than 0.05 was considered statistically significant.

We fitted multilevel mixed-effects logistic regression models with workplace specified as a random intercept to account for clustering. Models were estimated using restricted maximum likelihood (REML). Random slopes for key predictors were tested but did not improve model fit and led to convergence issues; therefore, random intercepts were retained. Physical activity data were cleaned according to IPAQ-SF scoring guidance and internal quality checks: records with implausible values (e.g., ≥ 240 min/day in any activity category or ≥ 16 h/day total) and records with incomplete/missing key fields were excluded. Of 4,886 recruited participants, 889 were excluded, resulting in a final analytic sample of 3,997.

Adjusted odds ratios (ORs) and 95% confidence intervals (CIs) were obtained from multivariable logistic regression models with all covariates entered simultaneously (enter method). Each OR reported in Table 2 represents the adjusted association between the predictor and physical activity level relative to the reference category.

Continuous variables are presented as mean ± standard error (SE), and categorical variables as frequencies and percentages.

Analyses were performed on SPSS 24 (IBM Corporation, NY, USA, 2016).

## Results

A total of 4,886 employees participated in the EHCSIR, of whom 3,997 individuals were included in the final analysis after excluding cases with missing data. The mean age of participants was 42.7 years; 35.9% were male and 64.1% were female.

Table 1 presents the mean and standard error (SE) of physical activity (PA) across key socio-demographic and occupational subgroups. The average total PA (sum of mild, moderate, and vigorous activity) was 722.2 MET-min/week, with a wide range (0 to 18,504). While, the average of moderate plus vigorous PA was 419.9 (< 600 MET-min/week). Higher PA levels were observed among males, younger individuals, ancillary staff, and those employees at hospitals. Moreover, individuals in the lowest economic quintile (Q1) reported the highest total PA, suggesting an inverse relationship between socioeconomic status and PA. Educational attainment and marital status also revealed distinct patterns: employees with lower levels of education and those who were married tended to report higher PA levels. Table 1 summarizes the PAL of the participants considering demographic data and socioeconomic status.

**Table 1:** Physical activity level (mean (SE)) of the participants in light of demographic and occupational variables (*N* = 3,997)

Demographic variables		Number	Intensity of PA			Total
			Mild	Moderate	Vigorous	
Age	< 34	619	382.01 (31.9)	169.04 (19.9)	355.6 (37.2)	906.7 (58.7)
	35-44	1,748	377.9 (19.4)	143.2 (10.7)	203.6 (17.5)	724.9 (30.5)
	45-54	1,286	400.5 (22.3)	122.7 (12.0)	131.8 (14.5)	655.1 (32.4)
	> 54	344	398.9 (42.5)	163.7 (38.5)	64.7 (17.3)	627.4 (66.1)
*p* value			0.877	0.189	< 0.001	**< 0.001**
Gender	Male	1,435	454.1 (25.0)	176.8 (14.7)	310.8 (22.7)	941.8 (40.1)
	Female	2,562	350.4 (14.0)	123.1 (8.4)	125.6 (10.9)	599.2 (21.5)
*p* value			**< 0.001**	**<0.005**	**< 0.001**	**< 0.001**
Marital status	Unmarried	538	305.6 (47.3)	134.4 (15.5)	211.4 (29.3)	651.5 (47.3)
	Married	3,213	397.9 (14.7)	144.8 (8.6)	191.2 (12.1)	733.9 (22.8)
	Ex-married	246	434.4 (51.7)	127.8 (33.6)	161.3 (44.8)	723.5 (82.3)
*p* value			**<0.05**	0.795	0.630	0.380
Wealth index	Q1	781	490.8 (37.1)	166.6 (21.4)	225.1 (27.3)	882.6 (55.9)
	Q2	820	425.7 (28.6)	150.4 (17.2)	196.1 (23.1)	772.3 (45.6)
	Q3	1,137	358.7 (21.0)	132.4 (12.3)	183.6 (20.6)	674.9 (34.9)
	Q4	676	318.7 (29.8)	120.1 (17.2)	144.8 (24.6)	583.8 (46.0)
	Q5	583	332.1 (24.4)	143.9 (17.7)	213.5 (26.5)	689.6 (41.3)
*p* value			**< 0.001**	0.384	0.217	**< 0.001**
Education	Elementary	400	593.2 (56.2)	205.0 (37.5)	179.7 (33.5)	977.9 (83.0)
	H.S. diploma	792	481.7 (36.1)	149.5 (18.5)	247.2 (29.3)	878.6 (55.6)
	Associate’s	376	387.7 (41.5)	118.9 (22.7)	257.7 (43.4)	764.5 (68.6)
	Bachelor’s	1,499	341.4 (17.8)	129.5 (10.6)	153.1 (15.7)	624.0 (27.8)
	Master’s	719	284.4 (18.8)	136.9 (15.7)	165.3 (20.6)	586.6 (35.7)
	PhD	211	325.2 (43.3)	148.9 (22.6)	260.2 (51.1)	734.4 (80.4)
*p* value			**< 0.001**	0.105	**<0.005**	**< 0.001**
Job	Office w.	1,276	307.2 (18.8)	119.4 (11.9)	166.2 (16.6)	592.8 (30.2)
	Faculty m.	135	448.6 (63.5)	191.1 (30.9)	318.8 (71.7)	958.6 (113.4)
	H.P.	1,809	357.9 (16.8)	123.5 (9.3)	147.2 (13.5)	628.7 (24.9)
	Ancillary s.	777	578.4 (40.0)	215.6 (25.0)	317.0 (34.5)	1111.2 (64.8)
*p* value			**< 0.001**	**< 0.001**	**< 0.001**	**< 0.001**
Workplace	Office	1,551	367.2 (20.8)	150.8 (12.4)	203.6 (18.0)	721.7 (34.0)
	H.C.	711	315.6 (21.9)	101.4 (12.5)	135.4 (22.1)	552.6 (36.2)
	Hospital	1,735	435.4 (20.7)	151.6 (12.3)	205.0 (16.8)	792.1 (31.4)
p value			**<0.005**	**<0.05**	0.053	**< 0.001**

*Bold Findings: statistically significant; Office w. *office worker, *faculty m.* faculty member, *H.P.* healthcare professional, *ancillary s.* ancillary staff, *H.C.* health center

Table 2 illustrates the distribution of participants by physical activity categories according to WHO guidelines. Overall, only 40.3% of males and 33% of females were adequately active, and 13.3% of males and 5.9% of females met the criteria for being highly active. In other words, 64.4% of the participants in our study did not engage in sufficient physical activity and were classified as physically inactive. Physical inactivity was most prevalent in individuals aged >54 years (68.6%), females (67%), married participants (64.7%), employees in Q4 of the wealth index (70.1%), associates (66.2%), office workers (67.6%), and workers in health centers (67.1%). In addition, the most highly active individuals were those younger than 34 years (15.3%), males (13.3%), unmarried participants (9.1%), those in Q1 of the wealth index (10%), those with a high school diploma (10.7%), ancillary staff (14.2%), and workers worked in offices (9.4%).

**Table 2:** Frequency and adjusted odds ratios (OR [95% CI]) of being adequately and highly active across demographic and occupational variables (*N* = 3,997)

Demographic variables	Adequately active	Adequately active(adjusted OR,95% CI)	Highly active	Highly active(adjusted OR, 95% CI)
Age				
<34	251 (40.5)	1	95 (15.3)	1
35-44	608 (34.8)	0.82(0.63, 1.08)	144 (8.2)	0.93(0.56,1.55)
45-54	458 (35.6)	0.85(0.65, 1.11)	83 (6.5)	0.66(0.40,1.07)
>54	108 (31.4)	**0.68 (0.50,0.91) **	21 (6.1)	**0.35(0.21,0.58)**
Gender				
Male	579 (40.3)	1	191 (13.3)	1
Female	846 (33.0)	**0.67 (0.57, 0.99) **	152 (5.9)	**2.08(1.61,2.70)**
Marital status				
Unmarried	199 (37.0)	1.28 (0.96,1.70)	49 (9.1)	1.02 (0.93,1.18)
Married	1,134 (35.3)	**1**	277 (8.6)	1
Ex-married	92 (37.4)	1.10 (0.79, 1.54)	17 (6.9)	0.65(0.52,0.77)
Wealth index				
Q1	290 (37.1)	1	78 (10.0)	1
Q2	304 (37.1)	0.98(0.31, 0.99)	72 (8.8)	**0.63(0.13,9.66)**
Q3	402 (35.4)	**0.62(0.29,1.42)**	94 (8.3)	**0.46(0.02,0.70) **
Q4	202 (29.9)	**0.44(0.47, 1.39)**	41 (6.1)	**0.53(0.04,0.95) **
Q5	227 (38.9)	0.53(0.32-0.81)	58 (9.9)	1.06(0.98,1.18)^#^
Education				
Elementary	158 (39.5)	1	41 (10.3)	1
H.S. diploma	285 (36.0)	0.93(0.53, 0.96)	85 (10.7)	1.13(0.89,1.37)
Associate’s	127 (33.8)	**0.65(0.24, 0.84)**	35 (9.3)	0.87(0.65,1.03)
Bachelor’s	518 (34.6)	**0.77(0.41,0.92)**	104 (6.9)	0.56(0.43,0.86)
Master’s	252 (35.0)	0.91(0.84,0.99)	58 (8.1)	0.87(0.75,1.14)
PhD	85 (40.3)	0.96(0.87, 1.04)	20 (9.5)	0.93(0.82,1.25)
Job				
Office w.	67 (5.2)	1	26 (2.3)	1
Faculty m.	43 (32.4)	2.13(1.91,2.78)	15 (11.1)	1.49(1.06,2.09)
H.P.	622 (34.4)	**2.91(1.58, 3.03)**	122 (6.7)	1.36(0.70,2.66)
Ancillary staff	322 (41.4)	**3.19(2.15,3.78)**	110 (14.2)	**1.54(1.09,2.17)**
Workplace				
Office	541 (34.9)	1	146 (9.4)	1
H.C.	234 (32.9)	0.71 (0.45,0.99)	39 (5.5)	0.44(0.39,1.34)
Hospital	650 (37.5)	1.32 (0.88,1.76)	158 (9.1)	**0.92(0.55,1.24)**

Bold findings: statistically significant (*p* < 0.05); # *p* = 0 .06. All odds ratios shown are adjusted values obtained from a multivariable logistic regression model including all covariates simultaneously. Distribution of participants by physical activity categories according to WHO guidelines. ‘Adequately active’ defined as ≥600 MET-min/week based on IPAQ scoring

*Office w.* office worker, *faculty m.* faculty member, *H.P.* healthcare professional, *H.C.* health center

Office w.: office worker; faculty m.: faculty member; H.P.: healthcare professional; H.C.: health center; Bold findings: statistically significant (*p* < 0.05); # *p* = 0 0.06. All odds ratios shown are adjusted values obtained from a multivariable logistic regression model including all covariates simultaneously. Distribution of participants by physical activity categories according to WHO guidelines. ‘Adequately active’ defined as ≥ 600 MET-min/week based on IPAQ scoring.

Table 3 presents mean PAL by gender and wealth index quintiles. Among males, those in Q1 had the highest activity levels across all domains. In contrast, female participants from Q4–Q5 demonstrated greater levels of moderate and vigorous PA. Mild PA (e.g., walking) among females was most prominent in Q1, while structured physical activities such as different sports were more common in wealthier female subgroups.

**Table 3:** Mean (SE) of physical activity in terms of the wealth index (Q1-5) and gender (*N* = 3,997)

		*N*	Intensity of PA			Total
			Mild	Moderate	Vigorous	
Q1	Male	383	556.0 (58.9)	220.0 (38.2)	336.7 (45.3)	1,112.8 (92.3)
	Female	398	428.0 (45.5)	115.1 (20.0)	117.8 (30.3)	661.0 (62.7)^*^
Q2	Male	334	493.5 (55.3)	206.8 (33.5)	331.0 (44.8)	1,031.4 (87.2)
	Female	486	379.1 (29.6)	111.6 (17.4)	103.5 (23.0)	594.3 (46.2)^*^
Q3	Male	399	447.0 (43.3)	141.0 (18.2)	306.4 (45.1)	894.5 (68.9)
	Female	738	311.0 (22.1)	127.8 (16.2)	117.2 (20.0)	556.1 (38.1)^*^
Q4	Male	180	277.7 (48.9)	95.3 (18.3)	272.0 (67.7)	645.0 (91.7)
	Female	496	333.6 (36.6)	129.1 (22.4)	98.7 (22.6)	561.5 (53.2)^*^
Q5	Male	139	327.5 (51.6)	193.9 (47.8)	253.8 (59.9)	775.2 (90.7)
	Female	444	333.6 (27.7)	128.2 (17.8)	200.9 (29.3)	662.7 (46.1)^*^

*Significant difference (*P* <0.05)

Results from the multilevel logistic regression models (Table 2) identified younger age, male gender, and Q1 wealth index as significant predictors of adequate activity. Occupational category was also strongly associated; ancillary staff, faculty members, and healthcare professionals were more likely to meet WHO recommendations compared to office workers. Workplace was a notable contextual factor, with employees at health centers showing the lowest likelihood of being sufficiently or highly active.

Mixed-effects linear regression analysis revealed significant associations between several socio-demographic variables and total physical activity (Table 4). Odds ratios (OR) and 95% confidence intervals (CI) are reported with two decimal precision. All values were reviewed for consistency and accuracy. No multicollinearity was detected among predictors. Male participants exhibited notably higher physical activity levels compared to females (β = 217.8; 95% CI: 130.5 to 305.1), and individuals who were unmarried also demonstrated increased activity compared to married individuals (β = 125.3; 95% CI: 103.1 to 176.2). Advancing age was inversely associated with physical activity, with the most substantial reductions observed in participants aged 45–54 and ≥ 55 years. An inverse association was noted between educational level and physical activity, indicating that individuals with advanced degrees may engage in less physical activity than those with lower formal education. Occupation type showed marked disparities; ancillary staff reported significantly higher activity than administrative employees (β = 391.6; 95% CI: 266.1 to 517.1). Additionally, employees working at hospitals or healthcare centers were more physically active than those in administrative offices. Based on Table 4, while employees in higher wealth quintiles (Q2 to Q5) showed lower average physical activity compared to those in Q1, the differences were not statistically significant because all confidence intervals included zero.

**Table 4:** Socio-demographic factors associated with total physical activity (in MET-minutes / week) (*N* = 3,997)

	Total MET-minutes(95% CI)
Intercept	829.58 (636.83, 1022.35)
Age	
< 34 (ref)	
35-44	-138.07 (-256.95, -19.19)
45-54	-225.5 (-344.33, -106.61)
> 54	-267.2 (-431.71, -102.60)
Gender	
Female (ref)	
Male	217.8 (130.55, 305.12)
Marital status	
Married(ref)	
Unmarried	125.3 (103.16, 176.21)
Ex-married	-32.3 (-45.26, 23.42)
Wealth index	
Q1 (ref)	
Q2	-35.4 (-156.51, 85.73)
Q3	-83.02 (-198.37, 32.32)
Q4	-61.3 (-196.03, 73.48)
Q5	-34.05 (-173.42, 105.26)
Education	
Elementary (ref)	
H.S. diploma	-16.3 (-34.54, -4.24)
Associate’s	-23.5 (-45.63, -9.23)
Bachelor’s	-88.4 (-105.62, -23.92)
Master’s	-39.6 (-103.7, -76.35)
PhD	-11.4 (-89.8,-45.8)
Job	
Office w. (ref)	
Faculty m.	32.02 (-209.91, 273.94)
H.P.	63.2 (-41.06, 167.52)
Ancillary staff	391.6 (266.17, 517.12)
Workplace	
Office (ref)	
H.C.	398.4 (254.21, 478.90)
Hospital	276.4 (189.18,378.22)

Results from mixed-effects linear regression analysis of socio-demographic predictors of physical activity. ‘Adequately active’ defined as ≥600 MET-min/week according to IPAQ scoring protocol

*Office w.* office worker, *faculty m.* faculty member, *H.P.* healthcare professional, *H.C.* health center

After data cleaning and removal of incomplete records, 889 participants were excluded, yielding a final sample of 3,997. The intraclass correlation coefficients (ICC) for workplace clustering ranged from 0.04 to 0.11, indicating that between-workplace differences accounted for a modest proportion of variance in physical activity outcomes. Sensitivity checks confirmed that the direction and magnitude of the main associations were robust to the exclusion of extreme and incomplete cases, although mean total MET-min/week decreased as expected after cleaning.

**Fig. 1:**
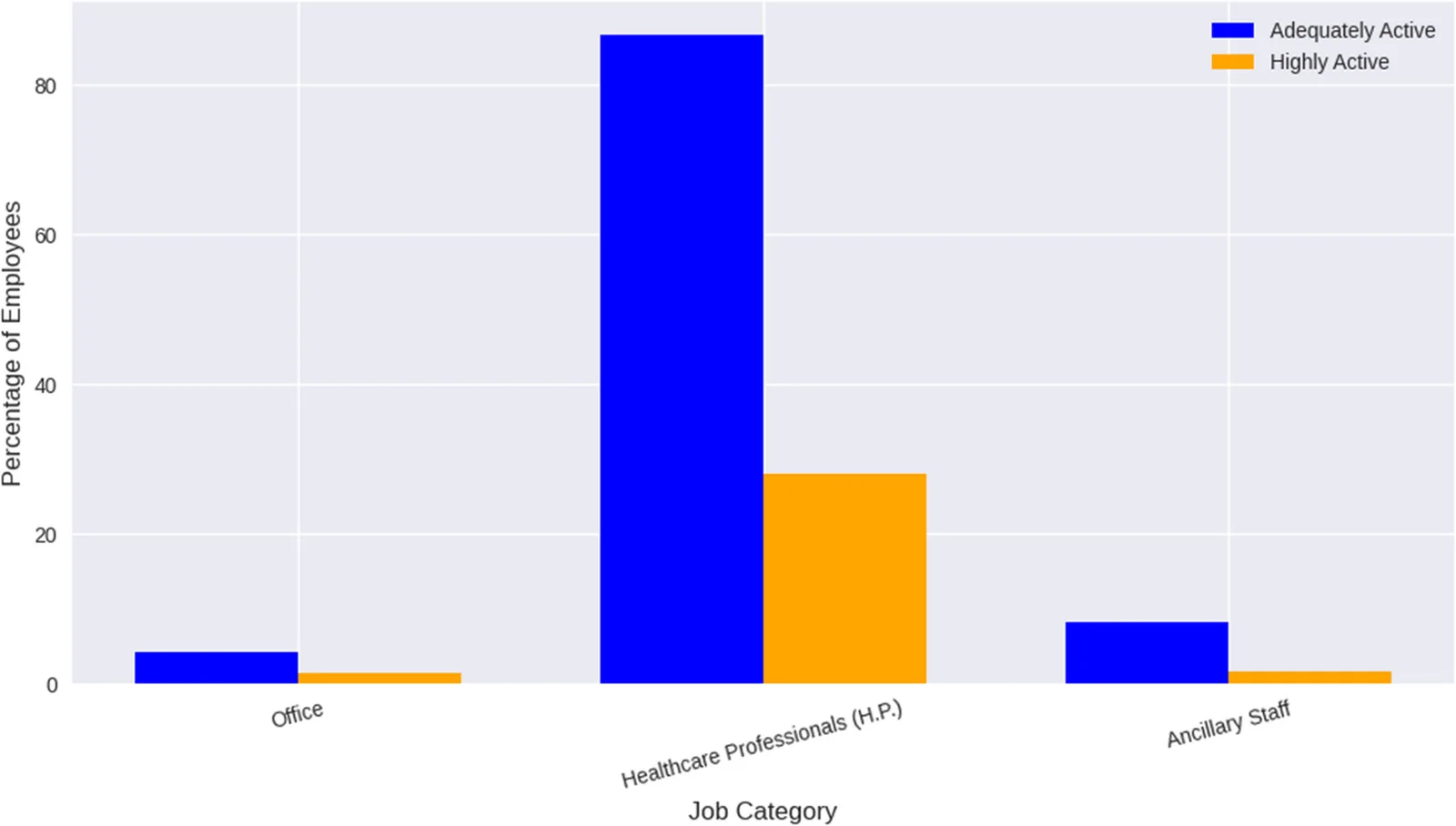
Physical activity levels by job category

Figure 1 illustrates the distribution of total PA across the job categories included in the study. Healthcare professionals accounted for the largest proportion of both adequately active and highly active employees, while office and ancillary staff represented smaller proportions. Considerable heterogeneity in physical activity patterns supports the inclusion of workplace as a random effect in regression models to account for clustering.

## Discussion

This study was guided by the social determinants of health framework and the ecological model of physical activity, both emphasizing the influence of socioeconomic position, job demands, and environmental context on health behaviors. Our findings clearly reflect these frameworks, demonstrating that occupational setting, job type, and socioeconomic gradients meaningfully shape physical activity patterns among Iranian employees.

We found a strong association between occupational status and PAL, with office employees and healthcare personnel working in health centers exhibiting the lowest levels of activity. Younger employees (< 34 years) reported higher total and vigorous activity, consistent with findings among American adults [30], Iranian workers [31], and Chinese employees [32]. This pattern likely reflects higher physical fitness and motivation among younger individuals, although cross-sectional data cannot establish causality. Cultural norms, varying fitness levels, and differences in lifestyle across populations may further explain inconsistencies with studies such as that among Austrian bank employees, which reported no age-related differences [33].

Gender differences were substantial. Men reported higher total, moderate, and vigorous PA, consistent with evidence from Iran and other countries [34–45]. Women, however, reported more mild PA. Notably, women in the Q4 wealth group—mostly basic-science faculty with fewer administrative responsibilities—engaged more in mild activity, likely reflecting greater discretionary time for walking.

Unmarried employees were more likely to meet PA recommendations (AOR = 1.28, 95% CI: 1.06–1.43). Although an American study similarly reported higher PA among married adults [46], other studies have observed lower PA [31] or no significant differences [32]. These discrepancies may reflect varying cultural expectations, household roles, or social support structures across settings.

Socioeconomic gradients in PA were pronounced. Employees in the lowest wealth quintile (Q1) exhibited the highest overall activity, likely due to physically demanding jobs, while individuals in higher wealth groups, particularly Q4, were less likely to meet WHO recommendations. Interestingly, both Q1 and Q5 showed elevated proportions of high PAL, although for different reasons. In the absence of domain-specific IPAQ data, previous research provides context: higher‐SES individuals in Iran demonstrate greater leisure-time PA [31], and Chinese civil servants show high levels of work‐related walking [32]. These variations reinforce the influence of cultural and occupational structures on PA patterns.

Lower educational attainment was also associated with higher overall PAL, consistent with more physically demanding occupations among less formally educated employees and with prior Iranian findings [31]. Conversely, studies in China reported higher PA among more educated civil servants [32], reflecting distinct occupational and cultural profiles. In our sample, higher-SES and more educated employees engaged in more vigorous PA, likely driven by leisure‐based activities, while lower‐SES employees accumulated more work‐related activity. This pattern aligns with previous work suggesting that motivations and opportunities for PA differ across social groups [18–21, 47].

Occupational classification further explained disparities in activity: ancillary staff, especially those in hospitals, reported the highest PAL due to manual job demands and larger spatial layouts that require constant movement. Office workers, in contrast, exhibited the lowest vigorous PA, reflecting sedentary job structures, a pattern consistent with earlier studies [48–50]. Healthcare personnel in health centers reported lower activity than their hospital counterparts, paralleling observations by Shahlaee and Nasiri [51], who noted more physically demanding responsibilities among hospital staff.

Across all demographic and occupational categories, mild PA was the most frequently reported activity. This is expected given IPAQ criteria, where walking is widely accessible and does not require special preparation or equipment. Vigorous PA was more commonly reported among men, younger employees, and faculty members, echoing findings among university students who typically have better fitness, higher motivation, and greater access to sports activities [52].

Despite these patterns, the overall level of moderate-to-vigorous PA was low. Participants accumulated an average of 419.9 MET-min/week, below the WHO-recommended threshold of 600 MET-min/week [53–57]. This is markedly lower than that reported among university employees in the UK (1,770 MET-min/week) [58], Chinese civil servants (2,227 MET-min/week) [32], and the Iranian general population (1,192–1,573 MET-min/week) [59]. Compared globally, the PA levels in our sample are approximately half of those reported in Poland (1,500 MET-min/week) and one third of those in Norway (> 2,200 MET-min/week) [60]. The high prevalence of inactivity (64.4%) observed here mirrors patterns among employees elsewhere, including Italian office workers (36.9% inactive) [61] and German workers (39.2% with no sport participation) [62].

These low levels of activity may be attributed to workplace barriers such as heavy workloads, limited time, lack of facilities, and insufficient organizational support. Qualitative research among Iranian healthcare workers highlights similar structural and individual obstacles, including time constraints and inadequate motivation [63]. Sedentary occupational structures—particularly in office environments—are major contributors, as workers spend approximately 60–70% of waking hours at work, with over 75% of that time sitting [64–66]. In large metropolitan areas such as Tehran, long commuting times and financial pressures (e.g., moonlighting) further restrict opportunities for PA.

These findings underscore the urgent need for organizational and policy-level interventions. Effective strategies may include improving workplace design (e.g., walkable environments, accessible staircases, and exercise facilities), integrating short movement breaks into sedentary jobs, offering flexible work schedules that allow PA during or after working hours, promoting active commuting, and developing tailored programs for high-risk groups such as office workers and health center staff. Longitudinal research and the use of domain-specific measures—such as IPAQ-Long Form or accelerometers—are needed to clarify causal pathways and more accurately capture the contributions of occupational, transport, and leisure domains.

Regarding data reduction, the decrease from 4,886 to 3,997 participants resulted from protocol-based IPAQ-SF cleaning and exclusion of incomplete records, thereby improving internal validity without affecting the overall patterns of association.

### Strengths and limitations

This study provides a comprehensive assessment of walking, moderate-intensity, and vigorous physical activity among Iranian employees, using total weekly MET-minutes, to evaluate adherence to recommended PAL. A strength of the present study is the occupational diversity and large size of its sample, with nearly 4,000 employees from various occupational and socioeconomic backgrounds, the findings are broadly generalizable across workplace settings. Other notable strengths offered by this study include using a standardized instrument such as validated short-form IPAQ; Robust socioeconomic analysis in which wealth index was derived using principal component analysis (PCA), allowing for nuanced stratification of SES-related patterns; and focused examination of gender and workplace effects.

The study also highlights key disparities in physical activity linked to gender roles and job environments, offering actionable insights for workplace health promotion.

This study has several limitations that should be considered:

- Self-reported physical activity was assessed using the IPAQ, although is widely recognized as the method of choice for assessing PA in epidemiological research [67], it may be subject to recall and social desirability bias.
- Sedentary behavior was not measured, limiting our ability to assess total movement patterns and inactivity risks.
- The cross-sectional design prevents causal inference between physical activity and demographic or occupational factors.
- We did not separate physical activity by domain (e.g., leisure, transport, occupational), which may obscure contextual differences in activity patterns.
- Future studies should incorporate objective measures of activity —such as accelerometers or wearable sensors—to improve accuracy and reliability, assess sedentary time, and explore domain-specific behaviors using longitudinal designs.

## Conclusion

This study identified significant associations between physical activity levels and demographic, socioeconomic, and occupational factors among Iranian employees. The findings highlight in one hand, disparities in physical activity, particularly among older adults, women, office workers, and higher-SES groups and on the other hand, approximately 64% of the employees taking part in this study did not meet the levels of PA recommended by the WHO. These patterns suggest that workplace context and job type play a critical role in shaping employee health behaviors. While the cross-sectional design limits causal inference, the results underscore the need for targeted interventions and policy strategies to promote physical activity in diverse occupational settings.

## Data Availability

The datasets used and analyzed during the current study are available from the corresponding author upon reasonable request.
